# Chitosan encapsulation modulates the effect of capsaicin on the tight junctions of MDCK cells

**DOI:** 10.1038/srep10048

**Published:** 2015-05-13

**Authors:** M. Kaiser, S. Pereira, L. Pohl, S. Ketelhut, B. Kemper, C. Gorzelanny, H. -J. Galla, B. M. Moerschbacher, F. M. Goycoolea

**Affiliations:** 1 Institute of Plant Biology and Biotechnology (IBBP), Westfälische Wilhelms-Universität Münster, Schlossgarten 3, Münster 48149, Germany; 2 Biomedical Technology Center of the Medical Faculty, Westfälische Wilhelms-Universität Münster, Mendelstraße 17, Münster 48149, Germany; 3 Experimental Dermatology, Department of Dermatology, Medical Faculty Mannheim, Heidelberg University, Theodor-Kutzer-Ufer 1-3, Mannheim 68167, Germany; 4 Institute for Biochemistry, Westfälische Wilhelms-Universität Münster, Wilhelm Klemm Straße 2, Münster 48149, Germany

## Abstract

Capsaicin has known pharmacological effects including the ability to reversibly open cellular tight junctions, among others. The aim of this study was to develop a strategy to enhance the paracellular transport of a substance with low permeability (FITC-dextran) across an epithelial cell monolayer via reversible opening of cellular tight junctions using a nanosystem comprised by capsaicin and of chitosan. We compared the biophysical properties of free capsaicin and capsaicin-loaded chitosan nanocapsules, including their cytotoxicity towards epithelial MDCK-C7 cells and their effect on the integrity of tight junctions, membrane permeability and cellular uptake. The cytotoxic response of MDCK-C7 cells to capsaicin at a concentration of 500 μM, which was evident for the free compound, is not observable following its encapsulation. The interaction between nanocapsules and the tight junctions of MDCK-C7 cells was investigated by impedance spectroscopy, digital holographic microscopy and structured illumination fluorescence microscopy. The nanocapsules modulated the interaction between capsaicin and tight junctions as shown by the different time profile of trans-epithelial electrical resistance and the enhanced permeability of monolayers incubated with FITC-dextran. Structured illumination fluorescence microscopy showed that the nanocapsules were internalized by MDCK-C7 cells. The capsaicin-loaded nanocapsules could be further developed as drug nanocarriers with enhanced epithelial permeability.

Many animal tissues are covered with sheets of epithelial or endothelial cells that are connected via proteinaceous tight junctions to create a network. These networks play a key role in the mechanical properties of tissues and also facilitate protection against pathogens^1^,^2^. Drugs must overcome this type of biological barrier to reach their target tissues and exert therapeutic effects. Many synthetic nanoparticle formulations for targeted drug delivery have been described, but researchers have focused more recently on biologically-derived materials such as polysaccharides, proteins and plant-derived natural compounds as components of nanoformulations that are fully biodegradable, biocompatible and nonimmunogenic, therefore providing attractive candidates for the development of innovative therapeutic delivery strategies^3^.

Chitosan is a natural aminopolysaccharide comprising glucosamine and N-acetyl glucosamine units. This biopolymer is widely used for the development of biomedical nanoformulations and shows diverse biological activities towards mammalian cells, including mucoadhesion^4^, the ability to condense and transport oligonucleotides^5^, and adjuvant activity^6^. Chitosan in solution or in the form of nanoparticles can also influence the integrity of tight junctions, opening them *in vitro*^7^,^8^,^9^,^10^,^11^,^12^,^13^,^14^ and *in vivo*^13^ to achieve enhanced paracellular permeability.

Capsaicin is the pungent vanilloid compound in spicy chili peppers that in mammals is known to interact with the TRPV1 receptor, an ion channel responsible for heat sensing^15^. Capsaicin has numerous therapeutic applications including the regulation of body temperature, the treatment of chronic pain and the treatment of obesity^16^. At a cellular level, capsaicin causes the reversible opening of tight junctions^17^,^18^, and the molecular basis of this phenomenon was recently investigated in MDCK cells^19^. However, the administration of capsaicin is not always feasible due to its pungency, cytotoxicity at high concentrations^20^, and sparing solubility in water^21^. Several studies have been conducted to incorporate capsaicin into nanoformulations in an attempt to make it more compatible with aqueous physiological environments^22^,^23^,^24^.

Colloidal nanocapsules with an oily core and a chitosan shell have been investigated as potential nanocarriers for transmucosal drug delivery^25^. These systems assemble by spontaneous emulsification 26,27 and are versatile because they can carry both lipophilic and hydrophilic macromolecules^26^,^28^,^29^,^30^,^31^,^32^.

The aim of the present study was to develop a nanocarrier system based on chitosan nanocapsules to attenuate the adverse properties of free capsaicin (cytotoxicity and sparing solubility) while preserving its ability to modulate tight junctions. This approach should enhance the paracellular transport of a co-administered substance with low permeability as an innovative design for a drug carrier. We compared the physicochemical and biological activities of free capsaicin and capsaicin-loaded nanocapsules using epithelial MDCK-C7 cells as a model of tight junction integrity with strong barrier properties^33^,^34^. We carried out cytotoxicity assays and monitored changes in tight junction and barrier properties using different forms of microscopy and automatic impedance spectroscopy^35^. We found that in capsaicin-loaded nanosystems the cytotoxic response of MDCK-C7 cells to capsaicin is not observable at a concentration of 500 μM. Furthermore, the effect of capsaicin on tight junctions is modulated by encapsulation, without affecting its ability to enhance paracellular permeability for macromolecules.

## Results

### Nanocapsules and nanoemulsions have distinct physicochemical properties

**Figure 1:**
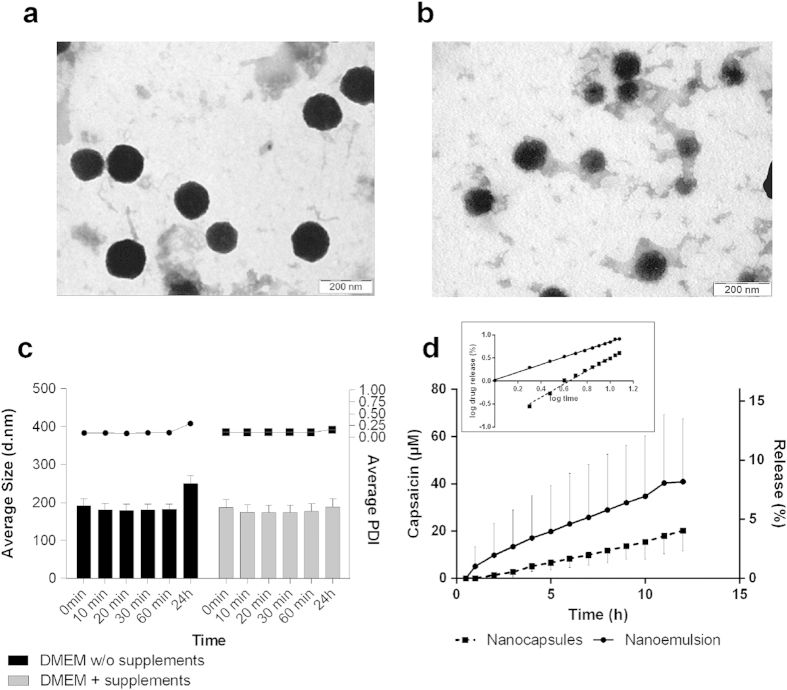
Representative TEM images of (**a**) nanoemulsions and (**b**) nanocapsules. (**c**) Evolution of diameter (bars) and average polydispersity index (PDI, line) of nanocapsules during incubation in DMEM cell culture medium (37 °C) with (black) and without (gray) supplements. (**d**) *In vitro* capsaicin release in MEM cell culture medium (37 °C) with a universal concentration of 500 μM capsaicin in all formulations. Data are mean values ± SD (*n* = 3). The inset shows the double logarithmic representation of the data.

The size, zeta potential and capsaicin-association efficiency of the nanoformulations are presented in Table 1 and agree with previously reported values^26^. The Z-average diameter of each formulation ranged from ~160 to ~260 nm, representing a polydispersity index (PDI) of ~0.1 to ~0.2. The nanocapsules had a strongly positive zeta potential (~+60 mV) and strong capsaicin-association efficiency (~92%) whereas the uncoated nanoemulsions lacking chitosan had a strongly negative zeta potential (~–80 mV) and weaker capsaicin-association efficiency (~50%). Fig. 1a,b show representative transmission electron microscopy (TEM) images of the formulations, both of which have a spherical morphology with sizes within the range determined by dynamic light scattering with non-invasive back scattering (DLS-NIBS) (Table 1). TEM images of the nanocapsules revealed a core-shell structure including a thin surface with irregular topography (Fig. 1b). Figure 1c shows the time-evolution average size and polydispersity index (PDI) measurements of the nanocapsules incubated in Dulbecco’s modified Eagle’s cell culture medium (DMEM) with and without supplements. The size and PDI remained constant in both environments for 24 h, in agreement with our previous studies of chitosan nanocapsules^26^. An *in vitro* capsaicin release assay is shown in Fig. 1d. Capsaicin release into the cell culture medium followed a near-linear pattern in both formulations. After 12 h, the nanoemulsion released a greater amount of capsaicin into the medium (~40 μM, ~8%) compared to the nanocapsules (~20 μM, ~4%) but there was greater variability between replicates in the nanoemulsions as indicated by the larger error bars. Both release profiles (inset in Fig. 1d) fitted to the linearized functions (Eq. 1, see methods section) in a double logarithmic plot (R^2^ ≥ 0.99). The derived *n* slopes for the release of capsaicin from the nanoemulsions and nanocapsules were 0.82 ± 0.01 and 1.44 ± 0.05, respectively.

### Nanoformulations are less toxic towards MDCK-C7 cells than free capsaicin

**Figure 2:**
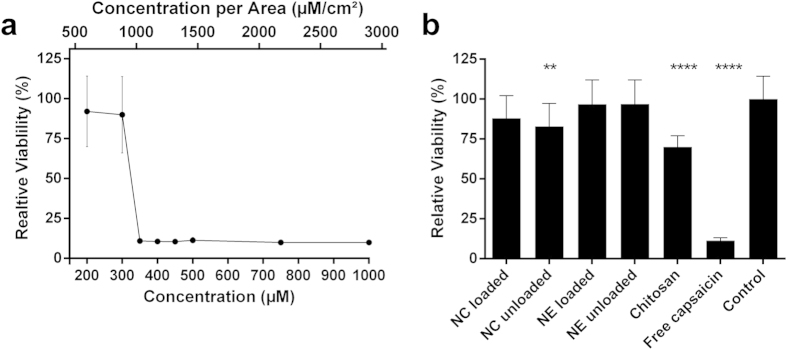
Cytotoxicity of different formulations against MDCK-C7 cells in 96-well plates determined using the MTT assay. (**a**) Relative cell viability following treatment with free capsaicin at increasing concentrations. Absolute concentrations are shown on the lower x-axis and concentrations per cultivated surface area are shown on upper x-axis. (**b**) Relative cell viability following treatment with the different nanoformulations or their constituents at a universal concentration of 500 μM capsaicin. For all experiments, cells were incubated for 3 h. Mean values ±SD. Statistical test: Kruskal-Wallis test (*n* = 3, ** p < 0.01, **** p < 0.0001). NC = nanocapsules; NE = nanoemulsion.

We investigated the influence of different concentrations of free capsaicin and the two nanoformulations and their constituents on the viability of MDCK-C7 cells, initially using an 3-(4,5-dimethylthiazol-2-yl)-2,5-diphenyltetrazolium bromide (MTT) assay to measure metabolic activity. Increasing concentrations of free capsaicin were evaluated over an incubation period of 3 h (Fig. 2a). A sharp drop in relative cell viability (from ~90% to ~10%) was observed over a narrow range of concentrations (~300 to ~350 μM) and this difference was statistically significant compared to the untreated control (p < 0.0001, Kruskal-Wallis test). At lower concentrations, the measuring errors (~20%) were more distinct than those observed within the cytotoxic concentration range. The cytotoxicity of the nanoformulations is shown in Fig. 2b, with a free capsaicin control included in the same panel for clarity. All formulations were tested at the same concentration of capsaicin (500 μM) and the treatments with loaded and unloaded nanoformulations were applied at the same carrier concentrations. The loaded nanoformulations did not significantly reduce cell viability compared to their unloaded counterparts at the same carrier concentrations. However, the chitosan polymer alone in solution at the same concentration used for the preparation of the nanoformulations caused a significant reduction in relative cell viability. The unloaded nanocapsules also had a small but significant impact on cell viability.

**Figure 3:**
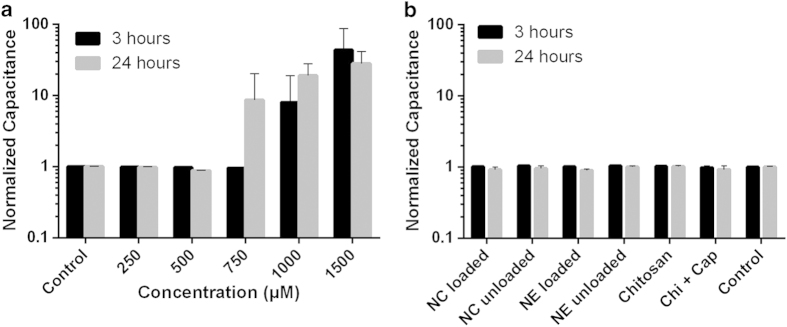
Capacitance measurements for cells grown on Transwell filters. (**a**) Normalized capacitance following treatment with free capsaicin at increasing concentrations. (**b**) Normalized capacitance following treatment with different nanoformulations or their constituents at a universal concentration of 500 μM capsaicin. Normalized capacitance is shown relative to the control for 3 h (black bars) and 24 h (gray bars) after the addition of capsaicin. Data are mean values ±SD (*n* = 3). NC = nanocapsules; NE = nanoemulsion.

We also estimated the cytotoxicity of the formulations by taking impedance measurements of capacitance (C_CL_) in MDCK-C7 cell monolayers grown on a porous filter membrane. C_CL_ has been used as indicator of cell viability in various experimental setups in previous studies^36^. Fig. 3a shows C_CL_ measurements at the 3 and 24 h time points as a function of the dose of free capsaicin. The C_CL_ begins to increase after 3 h at a concentration of 1000 μM, and after 24 h the same effect is also induced by 750 μM capsaicin. The all-or-nothing cytotoxicity response revealed by the C_CL_ measurements is similar to that observed in the MTT assay, although it occurs at a ~3-fold higher critical concentration (when compared after incubation for 3 h). Taking into account the cultivated surface areas in the two assays, both methods indicate that a capsaicin concentration of ≥900 μM/cm^2^ reduces cell viability significantly. Fig. 3b shows the C_CL_ measurements for the nanoformulations and their constituents with a constant a capsaicin concentration of 500 μM. No change in cell viability was observed in any of the treatments after 3 or 24 h.

### Loading nanoformulations with capsaicin increases their impact on trans-epithelial electrical resistance

**Figure 4:**
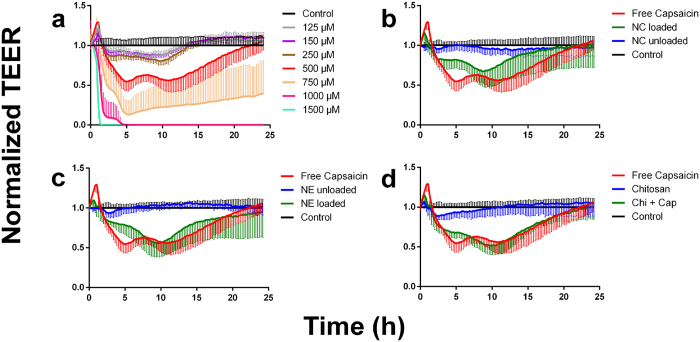
Impedance spectroscopy measurements to determine TEER. The normalized TEER relative to the control is shown over time: (**a**) Increasing concentrations of free capsaicin; (**b**) chitosan coated systems, (**c**) uncoated systems; (**d**) chitosan polymer. In (**b**), (**c**) and (**d**) the nanoformulations are compared with the behavior of free capsaicin. The nanoformulations an their constituents were applied at a universal concentration of 500 μM capsaicin. Data are mean values ±SD (*n* = 3). NC = nanocapsules; NE = nanoemulsion.

Figure 4a shows the time course of relative trans-epithelial electrical resistance (TEER) induced by increasing concentrations of capsaicin, whereas Fig. 4(b–d) show the comparative effect of the nanoformulations and some of their individual components. For clarity, the three latter plots show TEER values over time and are compared to the TEER behavior of the untreated control and free capsaicin at a concentration of 500 μM.

The free capsaicin plot (Fig. 4a) shows how the TEER profile depends on the concentration of capsaicin. Immediately after the addition of capsaicin, there was a sudden increase in TEER that peaked during the first ~20 min. This effect increased up to a concentration of 500 μM where it reached its maximum, and higher concentrations reduced the magnitude of this effect. After longer incubation times (up to 5 h), TEER decreased in a capsaicin dose-dependent manner. When considered over the entire time range, statistically significant differences in TEER values compared to the untreated control were observed at concentrations ≥500 μM (p < 0.0001, Friedman test). Approaching this concentration, the TEER curves showed two minima within the time ranges ~4-5 h and ~10–12 h, resembling a widened W shape, and recovered to approximately the original value after 24 h. If the concentration of capsaicin exceeded 500 μM, the cells were no longer able to recover to their original TEER value after 24 h, and at even higher doses of capsaicin the TEER values flattened out to zero. Close inspection of the traces representing the recovered cell monolayers revealed that the TEER values slightly exceeded their starting values after 24 h.

The corresponding TEER plot for the nanocapsules is shown in Fig. 4b. The unloaded nanocapsules induced a slight but significant decline in TEER (p ≤ 0.0001, Friedman test) after 2 h and between ~7 and ~18 h. The loaded nanocapsules showed an attenuated effect compared to the free capsaicin trace at the same concentration (p ≤ 0.0001, Friedman test). A close inspection of both traces showed that the initial increase in TEER was not as distinct as that observed in response to free capsaicin. This was followed by a sustained decline in TEER to reach a plateau. The first trough, which was observed in the free capsaicin trace at ~4 h, was not present in the nanocapsule trace, but a second decline began after ~5 h and reached a minimum after ~8 h. This was comparable to the second depression observed in the free capsaicin trace (minimum at ~12 h) albeit slightly earlier. The normalized minimum TEER values of the nanocapsules were not as extreme as those observed for free capsaicin. The TEER values in both the nanocapsule and free capsaicin treatments then recovered to their initial levels after 24 hours.

The TEER for the nanoemulsions is plotted in Fig. 4c. The unloaded nanoemulsions behaved in a similar manner to the unloaded nanocapsules, with only a moderate TEER effect. There was a small and statistically non-significant decline after 2 h (p > 0.05, Friedman test). The loaded nanoemulsions caused similar effect as the loaded nanocapsules (p ≤ 0.0001, Friedman test) and the intensity of the TEER depression was similar to that induced by free capsaicin.

The effect of the chitosan polymer on TEER is shown in Fig. 4d. The polymer induced a ~10% decline in TEER during the first ~10 h which was not statistically significant (p > 0.05, Friedman test), and beyond this point TEER recovered to the original value. The behavior of the physical blend of capsaicin and chitosan therefore differed from that of the capsaicin-loaded nanoformulations. The attenuation effect at the early stage of the experiment was still evident but the overall reduction in TEER was more pronounced (p ≤ 0.0001, Friedman test).

### Free capsaicin and capsaicin-loaded nanocapsules trigger the prolonged opening of tight junctions

**Figure 5:**
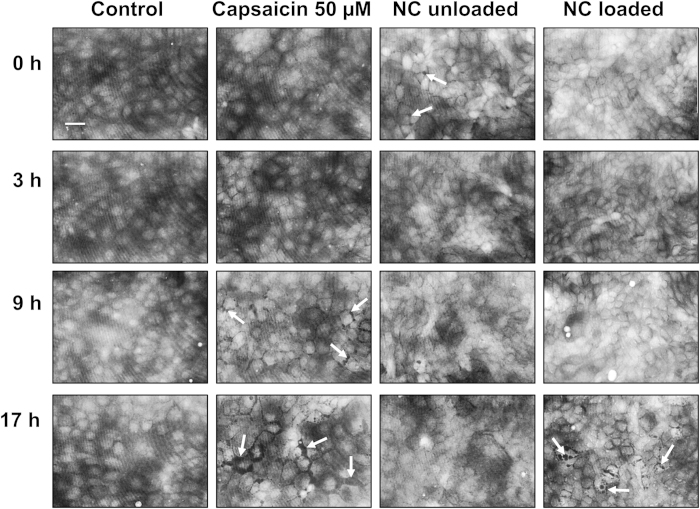
Representative DHM quantitative phase images of MDCK-C7 cell monolayers. From left to right: an untreated control, treatment with 50 μM free capsaicin, and treatments with unloaded and loaded nanocapsules (NC) at a concentration of 50 μM capsaicin, each at four different time points. Opening tight junctions are indicated by dark gaps in the quantitative phase contrast images (see arrows). Scale bar = 40 μm.

Figure 5 shows representative quantitative phase images of MDCK-C7 cell monolayers obtained with digital holographic microscopy (DHM) at several time points following treatment with 50 μM capsaicin, empty nanocapsules or capsaicin-loaded nanocapsules, plus untreated controls for comparison. The appearance of dark spots in the images (see arrows) indicated that tight junctions started opening 9 h after treatment with capsaicin. This effect became more pronounced after 17 h and persisted for even longer (data not shown). In contrast, no such spots were observed in the untreated control. Dark spots, indicating tight junction opening, were also observed when the cells were treated with nanocapsules. Interestingly, there was a near immediate response when cells were incubated with the unloaded nanocapsules whereas the capsaicin-loaded nanocapsules had a more pronounced effect on the tight junctions, reaching a peak at 17 h. As observed for free capsaicin, the tight junctions did not close even after more than 17 h exposure to the nanocapsules (data not shown).

### Free capsaicin and capsaicin-loaded nanocapsules increase the permeability of MDCK cell monolayers

**Figure 6:**
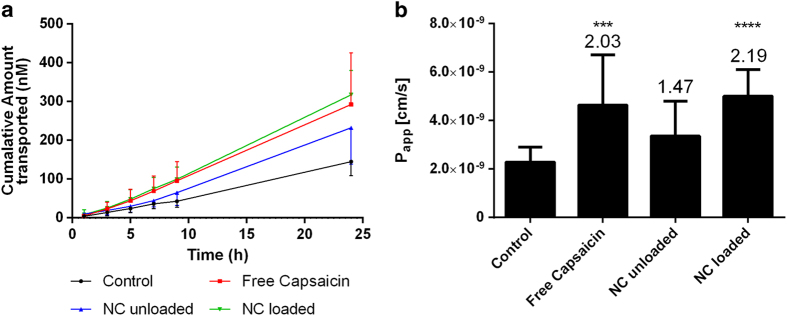
Permeability assay with MDCK-C7 cells and FITC-dextran (Mw = 4000 Da) following treatment with nanoformulations or controls at a universal concentration of 500 μM capsaicin. (**a**) Cumulative amount of dextran transported from the apical to the basolateral compartment over time after the beginning of treatment. (**b**) Permeability coefficient (P_app_) derived from the slopes of the transport curves. Enhancement factors are calculated with respect to the control. Data are mean values ± SD. Statistical test: Kruskal-Wallis test (*n* = 4, *** p < 0.001, **** p < 0.0001). NC = nanocapsules.

Next we carried out a permeability assay using fluorescein isothiocyanate (FITC)-labeled dextran with a molecular weight of 4000 Da as a model probe. The cumulative amount of labeled dextran transported across the MDCK-C7 cell monolayer increased linearly over time for all treatments including the control, although transport was most efficient when the cells were treated with 500 μM free capsaicin or the loaded nanocapsules (Fig. 6a). The variability in the amount of probe transferred in 24 h was lower for the loaded nanocapsules than free capsaicin, as shown by the smaller error bars. The unloaded nanocapsules also induced paracellular permeability, but not to the same extent as the loaded nanocapsules and free capsaicin. Fig. 6b shows the permeation coefficient (P_app_) values calculated from the slopes of the curves and the enhancement of permeability is shown as a relative enhancement factor calculated from the P_app_ ratio between each treatment and the control. These calculations indicated that free capsaicin and the capsaicin-loaded nanocapsules were able to increase the P_app_ by 2.0-fold and 2.2-fold respectively, compared to the control treatment, both of which were statistically significant increases. The unloaded nanocapsules caused a non-statistically significant ~1.5-fold permeability increase (p > 0.05, Kruskal-Wallis test) of the cells compared to the control cells.

### Loaded nanocapsules are internalized by MDCK-C7 cells

**Figure 7:**
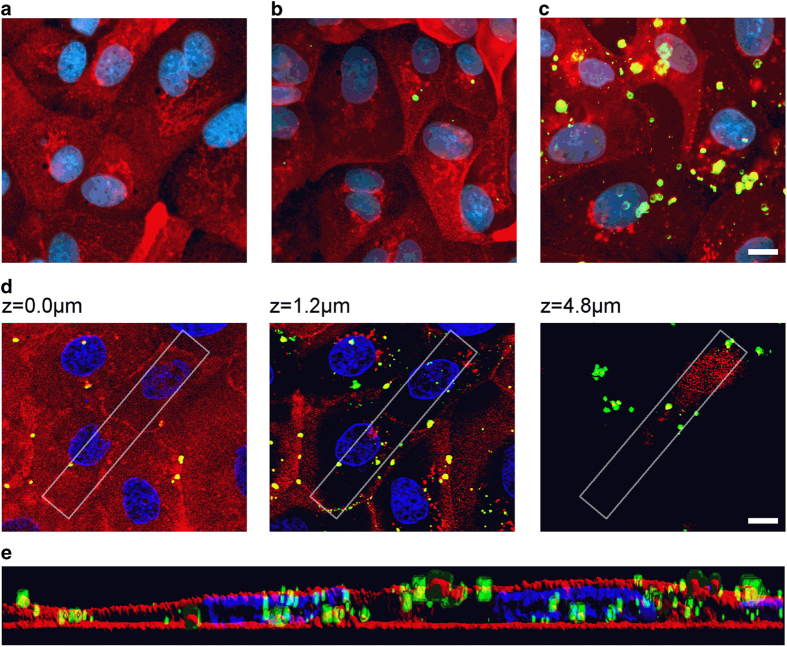
SIFM images of MDCK-C7 cells treated with capsaicin-loaded chitosan nanocapsules. MDCK-C7 cells remained untreated (**a**) or were treated with nanocapsules for 2 h (**b**) or 24 h (**c**). Scale bar = 10 μm. (**d**) Optical sections of the MDCK cell layer at the indicated heights (z-levels). Scale bar = 10 μm. White boxes mark the region of interest represented as a rendered three-dimensional vertical section (**e**). Nuclei were stained with DAPI (blue), chitosan nanocapsules were stained with CAP-sfGFP (green) and cell surfaces are stained with Texas Red-conjugated WGA (red).

Finally, we investigated the internalization of the nanocapsules by MDCK-C7 cells by three dimensional observation with structured illumination fluorescence microscopy (SIFM). The recently described chitosan-affinity protein fused to a superfold green fluorescent protein (CAP-sfGFP) was used for the specific detection of chitosan nanocapsules^37^ and the difference between attached and internalized nanocapsules was distinguished by counterstaining the membrane with wheat germ agglutinin (WGA) conjugated to Texas Red. Compared to untreated controls (Fig. 7a) only a few nanocapsules were attached to the cell surface after 2 h incubation (Fig. 7b) but this increased substantially after continuous co-culture for 24 h (Fig. 7c). A fraction of the formulation was also detected within the cells (Fig. 7d,e).

## Discussion

We used a combination of physical analysis, biological assays and microscopy to compare the ability of free capsaicin and capsaicin-loaded nanoformulations (comprising a lecithin-based oily core with or without a chitosan shell) to increase the permeability of tight junctions in MDCK-C7 cell monolayers. First we determined the physicochemical characteristics of the nanoformulations in terms of size, zeta potential, morphology, stability and capsaicin association efficiency (Table 1, Fig. 1) and found that our data agreed with previous reports^26^. We found that the chitosan-coated nanocapsules retained ~92% of the capsaicin cargo even when loaded with 800-fold more capsaicin than in these studies (10 vs 0.013 mM). In contrast, the nanoemulsions lacking chitosan showed a lower capsaicin-association efficiency (~50%), indicating that the presence of chitosan in the nanocapsule shell favors the retention of capsaicin during emulsification. Furthermore, we intended not to release capsaicin prior to its interaction with the cells but to achieve the permeability enhancement of a co-administered compound. The different nature of the shells in the two formulations was clearly demonstrated by the different *in vitro* capsaicin release profiles as discussed in more detail below.

We are aware that in the case of nanoemulsion only 50% of the capsaicin was associated and hence, we could have isolated the system to remove the free fraction of capsaicin. However, it is known that isolation leads to unavoidable experimental errors that might have resulted in an inaccurate estimation of the final concentrations of the formulation components. Moreover, since the *in vitro* release data (Fig. 1) showed only very moderate increase of released capsaicin concentration in the nanoemulsions with respect to nanocapsules, we opted to not isolate the formulations and preferred to keep track closely of the applied concentration of the components. This is consistent with recent studies by other groups using a similar nanosystem^32^. Due to the low association efficiency of capsaicin shown by the nanoemulsions, these systems were only used as a control for cytotoxicity and TEER studies. Nanocapsules, which associated more than 90% of capsaicin, were not isolated as the free fraction (~50 μM) is not sufficient to induce any significant effect on cytotoxicity (Fig. 2) and TEER (Fig. 4).

Cell viability studies based on MTT assays revealed that capsaicin is highly cytotoxic above a threshold concentration of ~300 μM, resulting in a sharp decline in metabolic activity. This may reflect cell swelling due to influx of ions (probably Ca^2+^) through the TRPV1 receptor, as reported in HEK293 cells^15^. A rapid influx of Ca^2+^ following exposure to capsaicin has also been reported in MDCK cells^38^. C_CL_ measurements, which can be used as a surrogate for cytotoxicity^36^, showed that cell viability was reduced following exposure of cells to free capsaicin at a concentration of ~1000 μM for 3 h or ~750 μM for 24 h. Taking the surface area covered with cells in the two assays into consideration, both methods showed that incubation with free capsaicin at concentrations ≥900 μM/cm^2^ for 3 h results in a substantial decline in cell viability. The cytotoxic dose of capsaicin (expressed in μM/cm^2^) may differ between the assays due to the different numbers of cells used in each case, which is determined by the diameters of the microwells in the 96-well microtiter plate and the polycarbonate membrane inserts. In turn, this may reflect the possibility that capsaicin is not fully dissolved in cell culture media, but is instead present as a microcrystalline phase that gradually sediments onto the cell surface^21^. The agreement between the results in the two assay techniques confirms that capacitance determined by impedance spectroscopy, although rarely used, is nevertheless a suitable indicator of cell viability.

The TEER of cells exposed to free capsaicin showed a reversible dose-dependent effect, in good keeping with previous reports^19^. This suggests that capsaicin could potentially be used as a natural permeability enhancer to open biological barriers. The TEER data were supported by DHM quantitative phase microscopy images, although after treatment with capsaicin the tight junctions remained open for the rest of the assessment period. This discrepancy may be explained by different cell culture conditions during live cell imaging with DHM. The permeability of the monolayer in the presence of FITC-dextran was also enhanced by capsaicin but the observed effect was two orders of magnitude less potent than reported elsewhere for MDCK cells^19^. This is reasonable because the C7 clone used in our study has much greater tight junction integrity than other clones of the same cell line^33^.

*In vitro* release studies (Fig. 1d) showed that the profile of capsaicin release fits a linear regression in a double logarithmic plot that allows the *n* exponent in Eq. 1 (see methods section) to be calculated at the early stages of release. The different values of the *n* exponent for each system reflect differences in the transport of capsaicin across the two types of nanoformulations. In microspheres^39^,^40^, it is known that the values of the *n* exponent are determined by the transport mechanism. The presence of chitosan on the surface of the nanocapsule resulted in a different release behavior compared to the nanoemulsions lacking a polymeric coat. The applied model (Eq. 1, see methods section) is reported to be valid up to 60% of the final weight of a released drug. As the R^2^ values were close to 1, we regarded this model suitable for the comparison of release profiles in both systems.

The capsaicin release profile of our formulations is distinct from that of many other nanosystems which release their payload in an uncontrolled manner, such as the biphasic burst like release profile observed for docetaxel^29^. The *in vitro* capsaicin release assays showed that both nanoformulations discharged capsaicin in a monotonic profile and that most of the drug (>90%) was retained during the time course of the TEER measurements. This is consistent with a model in which release is controlled by the partitioning of capsaicin between the components of the nanoformulations and the medium. It was also interesting to note that nanoemulsions released twice as much capsaicin as the nanocapsules. This emphasizes the role played by the polymer shell in the retention of capsaicin.

We found that, when encapsulated, capsaicin at a concentration of 500 μM can be applied without compromising the viability of MDCK-C7 cells compared the free molecule (Fig. 2). The chitosan shell itself does not appear to protect the cells directly against the effects of capsaicin because there was no significant difference between the nanocapsules and nanoemulsions. Significant reductions in cell viability were also observed when the cells were exposed to unloaded nanocapsules and the chitosan polymer. Whether this reflects a reduction in metabolic activity or genuine cell death is yet to be determined. The cytotoxicity of chitosan and chitosan-based nanoparticles has been documented^11^,^30^ and has been suspected to be due to the interaction between positively-charged residues on the chitosan polymer with negatively-charged glycocalyx on the cell surface, as reported for other polycationic reagents such as polyethyleneimines^41^,^42^. However, it is not clear why the capsaicin-loaded nanocapsules did not cause a significant reduction in cell viability when their unloaded counterparts induced a slight although statistically significant reduction in metabolic activity. Nevertheless, our data suggest that the encapsulation of capsaicin limits its cytotoxicity although the resulting changes in cell viability could not be detected by capacitance measurements.

The effect of the loaded and unloaded nanoformulations on the integrity of tight junctions was studied in more detail by measuring TEER behavior. The unloaded nanoemulsions had no significant impact on TEER, but the loaded nanoemulsions caused a substantial reduction in TEER even exceeding the effect caused by the loaded nanocapsules. The amount of free capsaicin present in loaded nanoemulsions is higher than that in the loaded nanocapsules due to the lower encapsulation efficiency. The stronger effect on TEER may therefore reflect the greater amount of free capsaicin in the system. The application of chitosan polymer solution caused a reduction in TEER consistent with previous studies in which chitosan solutions were applied to monolayers of Caco-2 cells^9^. Our data show unequivocally that capsaicin and capsaicin-loaded nanoformulations at the applied concentrations can cause the reversible disruption of tight junctions. Furthermore, the specific time profile of this effect can be modulated by the type of nanoformulation, and perhaps also by chitosan alone in solution as claimed elsewhere^43^.

To understand the significance of the TEER data, we conducted paracellular permeability assays using FITC-labeled dextran as a model hydrophilic macromolecule that crosses cell layers with low efficiency (Fig. 6). We found that although the unloaded nanocapsules had only a minor impact on TEER, they were nevertheless able to increase the amount of dextran transported across the cell layer albeit not to a statistically significant extent. The loaded nanocapsules were able to modulate the TEER of the cells, indicating that capsaicin activity persists when encapsulated. The low cytotoxicity induced by capsaicin even at higher doses of these formulations suggests that the high association efficiency and minimal release ensure that only small amounts of the free drug are available. In the permeability experiments, the loaded nanocapsules were observed to permeate the largest amount of dextran through the cell monolayer suggesting that nanocapsules can deliver capsaicin in a more efficient manner than the free drug applied in solution.

SIFM analysis showed that the chitosan nanocapsules accumulated on the cell surface and few were internalized during the assessment period (Fig. 7). A few nanocapsules were already localized at the cell–medium interface after incubation for 2 h, which matches the declining TEER values (Fig. 4b). The direct physical interaction between the nanocapsules and cell surface probably results in local particle enrichment to the extent that the concentration of free capsaicin is sufficient to open the epithelial barrier. Furthermore, the close proximity of the nanocapsules and the extracellular matrix components exposed by the cells may promote the release of capsaicin, increasing its availability in the cellular microenvironment. Mechanistic studies have to be carried out to elucidate the cellular uptake route and the intracellular effect of nanoencapsulated capsaicin.

DHM quantitative phase images (Fig. 5) confirmed that tight junctions opened in response to both the loaded and unloaded chitosan-coated nanoformulations. Chitosan and capsaicin promoted the opening of tight junctions at different time points, suggesting that different mechanisms are involved. The loaded nanoformulations delayed the opening of tight junctions compared to the individual effects of chitosan and capsaicin. A lower degree of cell stress was also observed, as shown by the less severe changes in morphology compared to the cells exposed to free capsaicin.

In conclusion, the nanoformulations we investigated may offer a novel strategy for the delivery of drugs across biological barriers using only natural compounds. Encapsulation substantially changes the behavior of capsaicin and higher concentrations of capsaicin can be applied as nanocapsules without inducing the level of cell stress caused by free capsaicin while achieving the same degree permeability. Further investigations will involve the co-loading of nanocapsules with capsaicin and other drugs to study their ability to deliver drugs across biological barriers. Our ongoing studies are currently addressing whether the encapsulation of capsaicin in nanoformulations also modulates its pungency.

## Materials and methods

### Chitosan

The sample of chitosan we used was an ultrapure biomedical grade Heppe 70/5 (Batch No. 212-140311-02) purchased from HMC^+^ GmbH (Halle/Saale, Germany). The molecular weight of 17,600 Da was determined by measuring its intrinsic viscosity in 0.3 M acetic acid/0.2 M sodium acetate at 25 °C^44^ and the degree of acetylation of 32.4% was determined by ^1^H NMR spectroscopy. Ultrapure MilliQ water was used throughout.

### Dissolution of capsaicin

Capsaicin (from *Capsicum* sp. 96%, Sigma-Aldrich, Steinheim, Germany) was dissolved in ethanol at a concentration of 24 mg/ml. For the cell culture experiments, this capsaicin stock was further diluted with modified Eagle’s medium (MEM) in a 50-ml Falcon tube. The medium was added with a pipette by dripping it gently along the wall of the inclined tube. As soon as the MEM mixed with the capsaicin solution the mixture became slightly turbid. These samples were immediately used without shaking. This protocol ensured reproducible results in the biological assays.

### Preparation of the nanoformulations

The chitosan-coated nanocapsules were prepared as previously described with slight modifications^26^. Briefly, 400 μl of a 100 mg/ml ethanolic lecithin solution (Epikuron 145 V, Cargill texturing solutions Deutschland GmbH & Co. KG, Hamburg, Germany) was mixed with 530 μl of the capsaicin stock solution described above (24 mg/ml). This was supplemented with 125 μl Miglyol 812 N (Sasol GmbH, Witten, Germany) and 9.5 ml acetone. The organic solution was immediately poured into 20 ml aqueous chitosan (0.5 mg/ml in 5% stoichiometric excess of 5 M HCl). The milky mixture was concentrated in a rotavapor (Büchi R-210, Büchi Labortechnik GmbH, Essen, Germany) at 50 °C until 3.5–4.0 ml remained and the volume was topped up to 4.0 ml with milliQ water if necessary to yield a final capsaicin concentration of ~10 mM. The nanoemulsions were prepared using the same procedure but without including chitosan. Unloaded nanocapsules and nanoemulsions were prepared by replacing the capsaicin solution with ethanol.

### Size and zeta potential

The size distribution of the nanoformulations was determined by dynamic light scattering with non-invasive back scattering (DLS-NIBS) with a measurement angle of 173°. The zeta potential was measured by mixed laser Doppler velocimetry and phase analysis light scattering (M3–PALS). A Malvern Zetasizer NanoZS (Malvern Instruments Ltd., Worcestershire, UK) fitted with a red laser light (λ = 632.8 nm) was used for both methods. The samples were diluted 1:50 in water before measurement.

### TEM

The ultrastructure of the nanoformulations was investigated by TEM using a CM12 instrument (Philips, Eindhoven, Netherlands). The samples were mounted on copper grids coated with Formvar® and stained with 10 mg/ml research grade uranyl acetate (Serva Electrophoresis GmbH, Heidelberg, Germany).

### High-performance liquid chromatography (HPLC) with UV detection

HPLC-UV was carried out using a Jasco HPLC system (Jasco GmbH, Gross-Umstadt, Germany) comprising a three-line degasser (DG-2080-53), a ternary gradient unit (LG-2080-02S), a semi-micro HPLC pump (PU-2085Plus), an autosampler (X-LC™ 3159AS), an intelligent column thermostat (CO-2060 Plus) equipped with a Kinetex C-18 reversed phase column (2.6 μm, C18, 50 × 2.1 mm, S/N 539947-37; Phenomenex, Torrance, USA) and a UV/Vis detector (X-L™ 3075UV). A mixture of 35% water and 65% acetonitrile was used in isocratic mode as the mobile phase at a flow rate was 0.15 ml/min. Capsaicin was detected at λ  = 228 nm.

### Association efficiency

The nanoformulations were partitioned by ultracentrifugation (Mikro 220 R, Hettich GmbH & Co. KG, Tuttlingen, Germany) at 16,000 rpm for 1 h at 15 °C, and the capsaicin content of the subnatant was determined by HPLC as described above. The association efficiency was calculated as the difference between the total amount of capsaicin incorporated in the formulation and the amount present in the subnatant.

### *In vitro* capsaicin release assay

An 800-μl aliquot of each formulation was transferred to a dialysis tube (Pure-a-lyzer Maxi 0.1–3.0 ml, Mw cut-off = 6 kDa, Sigma-Aldrich GmbH, Steinheim, Germany) and placed in a glass beaker containing 79.2 ml MEM previously equilibrated at 37 °C in an incubator. Every hour, a 500-μl aliquot of medium was removed and replaced with the same volume of MEM. The capsaicin content of the aliquots was determined by HPLC as described above. The transport of capsaicin from the nanoformulations into the medium was analyzed by fitting the data to the empirical equation^39^:
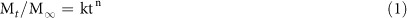
where M_t_ is the mass of capsaicin released at time t. The parameter M_∞_ represents the total mass of capsaicin to be released and k is a constant that depends on the structural characteristics of the nanoformulation and the solvent/material interactions.The exponent *n* is used to indicate the type of diffusion.

### Cell culture

Mandin Darby Canine Kidney (MDCK) cells clone C7^33^ were cultured in 75 cm^2^ flasks using MEM supplemented with 10% fetal bovine serum, 1% L-glutamine (200 mM) and 1% penicillin-streptomycin (10000 units penicillin, 10000 units streptomycin in 0.9% NaCl). The cultures were maintained in a humid atmosphere at 37 °C with 5% CO_2_ (Sanyo MCO-19AIC, Panasonic Biomedical Sales Europe BV, AZ Etten Leur, Netherlands). Cells from passages 22–34 were used for all experiments, which were carried out as independent triplicates on different days. After reaching microscopic confluence, the cells were washed with 10 ml phosphate buffered saline (PBS) and trypsinized with 10 ml 0.05% trypsin in EDTA (1x) buffer. After detachment, 10 ml of MEM was added to the trypsin buffer. The cell suspension was centrifuged at 1000 rpm for 5 min (Rotina 420 R, Hettich GmbH, Tuttlingen, Germany). The excess of medium was removed and the cell pellet was resuspended in 1 ml MEM. A 10-μl aliquot of the cell suspension was diluted with 90 μl trypan blue and the number of cells was counted with an improved Neubauer chamber before seeding. The cells were subcultured by splitting at a ratio of 1:10.

### 3-(4,5-dimethylthiazol-2-yl)-2,5-diphenyltetrazolium bromide (MTT) assay

The cytotoxicity of the nanoformulations and components was evaluated using an MTT assay^45^. Briefly, 100 μl of cell suspension was transferred to each well of a 96-well tissue culture plate (~10^4^ cells per well or ~10^5^ cells/ml) and allowed to attach for 24 h. The cells were washed twice with supplement-free MEM before the sample was added and the cells were incubated for 3 h. The samples were removed and replaced with 100 μl supplement-free MEM. We prepared an MTT solution in PBS with a concentration of 5 mg/ml of thiazolyl blue tetrazolium bromide and added 25 μl to each well. After 4 h, the medium was again removed and the dye was dissolved in DMSO. After orbital shaking at 300 rpm for 15 min, the absorbance was measured at λ = 570 nm in a microplate reader (Safire, Tecan AG, Salzburg, Austria). Relative viability values were calculated by dividing individual viabilities by the mean of the control. We used 4% Triton X-100 in PBS as a positive control.

### Electrical impedance measurements

The TEER and capacitance (C_CL_) of cell monolayers were measured using an automated CellZscope® instrument (nanoAnalytics, Münster, Germany). Approximately 1.5 ml of cell culture medium was transferred to the basolateral chamber of 12-mm Transwell Permeable Supports with 0.4-μm pore polycarbonate membrane inserts (Corning Inc., New York, USA). A 500-μl aliquot of MDCK cells in suspension was then seeded onto the membrane supports (~10^5^ cells per well) and allowed to grow for 4 days. Approximately 24 h before the experiment, every well was filled with 1.5 ml supplement-free MEM and the cell culture medium of the supports was replaced in the apical compartment with supplement-free MEM. The supports were transferred into the instrument and allowed to acclimate. After reaching a constant resistance of at least 5000 Ω cm^2^ the experiment was initiated by replacing 250 μl of the medium in the apical chamber with the sample dissolved in measuring medium at double the desired concentration. The resistance and capacitance was measured continuously for 24 h. The normalized TEER and capacitance values were calculated using Eq. 2:
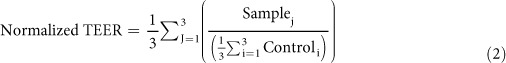


### Permeability assay

Cells were seeded at a density of 2.5 × 10^4^ cells per well on Transwell Permeable Supports as described above and provided with MEM lacking phenol red but supplemented like the medium used for the regular cell culture. The same volumes were used for the apical and basolateral chambers as described above. The cells were cultured for 4 days to develop confluent monolayers and the medium was replaced with serum-free transport medium and equilibrated overnight. The next day, TEER was measured in the Transwells. Only filters with an electrical resistance >5000 Ω cm^2^ were used. For these filters, half of the medium in the apical chamber was replaced with the same medium containing double the concentration of sample and 37.5 μl 50 mg/ml FITC-dextran, Mw 4000 Da, FITC:glucose 1:250; Sigma-Aldrich GmbH, Steinheim, Germany). Aliquots of medium (100 μl) were taken from the basolateral chamber after 1, 3, 5, 7, 9 and 24 h and replaced with fresh medium. The aliquots were transferred to 96-well plates (UV star black f-bottom, chimney well, μ clear; Greiner Bio-one GmbH, Frickenhausen, Germany) and fluorescence (λ_ex _= 485 nm, λ_em _= 520 nm) was measured in the microplate reader described above. P_app_ was calculated using Eq. 3 as previously described^46^:
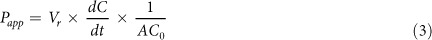
where V_r_ (cm^3^) represents the volume of the acceptor compartment, dC/dt (M/s) represents the slope of the cumulative concentration of the compound in the acceptor chamber over time, A (cm^2^) represents the membrane surface area and C_0_ (M) is the initial concentration of the compound in the donor chamber. The experiment was carried out as four independent replicates.

### Digital holographic microscopy (DHM)

Cells were seeded on microscope dishes (ibidi μ-Dish with glass lid, ibidi GmbH, Munich, Germany) in MEM at a density of 2.1 × 10^5^ cells/dish and were allowed to attach overnight. The following day the medium was replaced with MEM lacking supplements but containing the nanoformulations in 20 mM HEPES buffer. Quantitative imaging of MDCK cells by label-free DHM was carried out using an inverted microscope (iMIC, Till Photonics, Gräfelfing, Germany) with an attached DHM module^47^, and an incubator (Solent Scientific Ltd., Segensworth, UK) to ensure temperature stability. This instrument was used for bright-field imaging and quantitative DHM phase-contrast imaging. The coherent light source for the capture of digital holograms was a frequency-doubled neodymium-doped yttrium aluminum garnet (Nd:YAG) laser (Compass 315 M-100, Coherent, Lübeck, Germany, λ = 532 nm). Digital holograms of single cells were recorded continuously every 3 min using a 20x microscope lens (Zeiss LD Acroplan 20x/0.4 Korr). Quantitative phase images were reconstructed from the digitally-captured holograms by spatial phase shifting as previously described^48^,^49^ with custom-built software. Three independent measurements were taken in each experiment. Additional cell viability assays simulating the cell culture conditions during DHM analysis (including HEPES buffer but without a CO_2_-enriched atmosphere) showed a greater cytotoxic effect of capsaicin starting at a concentration of ~200 μM (see Supporting Information). We therefore chose a concentration of 50 μM capsaicin for these experiments.

### Structured illumination fluorescence microscopy (SIFM)

MDCK cells were seeded on glass slides and cultivated to confluence as described above. The cells were treated with 250 μM chitosan nanocapsules in supplement-free medium for 2 or 24 h. Prior to staining, the cells were fixed at 4 °C for 30 min in HEPES-buffered Ringer’s solution (10 mM HEPES, 5 mM glucose, 1 mM CaCl_2_, 1 mM MgCl_2_, 5 mM KCl, 140 mM NaCl) containing 4% paraformaldehyde. The cells were then permeabilized with incubation buffer (HEPES-buffered Ringer’s solution, supplemented with 0.3% Triton X-100 and 0.1% bovine serum albumin) and nonspecific staining was blocked with incubation buffer supplemented with 2% bovine serum albumin. Chitosan nanocapsules were stained with 100 μg/ml CAP-sfGFP and cell surfaces were counterstained with 2.5 μg/ml Texas Red conjugated to WGA (Life Technologies GmbH, Darmstadt, Germany). Nuclei were stained with 500 ng/ml 2-(4-amidinophenyl)-6-indolecarbamidine dihydrochloride (DAPI; Sigma-Aldrich GmbH, Steinheim, Germany). Coverslips were mounted in DABCO-Mowiol (Sigma-Aldrich GmbH, Steinheim, Germany) and analyzed by SIFM using an inverted fluorescence microscope (AxioObserver.Z1, Zeiss, Jena, Germany) equipped with a structured illumination module (ApoTome, Jena, Germany). Three-dimensional rendered images of optical sections were calculated with ImageJ^50^.

### Data analysis

Statistical analysis was carried out using Prism v6.0c (GraphPad Software Inc., La Jolla, USA). All experiments were statistically analyzed using non-parametric tests. The Kruskal-Wallis test was used for unpaired tests and the Friedman test was used for paired tests. All biological experiments were conducted at least in triplicate.

## Author Contributions

M.K., S.P., L.P., S.K. and C.G. conducted the experiments. M.K., S.P., C.G. and B.K. conceived the experiments. M.K. and F.M.G. wrote the manuscript. M.K. and L.P. analyzed the data. M.K. prepared Fig. 1, 2, 3, 4, 5, 6. C.G. prepared Fig. 7. B.M.M. and H.J.G. contributed to the interpretation of the experiments. All authors reviewed the manuscript.

## Additional Information

**How to cite this article**: Kaiser, M. *et al.* Chitosan encapsulation modulates the effect of capsaicin on the tight junctions of MDCK cells. *Sci. Rep.* **5**, 10048; doi: 10.1038/srep10048 (2015).

## Supplementary Material

Supplementary Information

## Figures and Tables

**Table 1:** Physicochemical properties of chitosan-coated nanocapsules (NC) and nanoemulsions (NE) with or without capsaicin (10 mM).

**Nanoformulation**	**Size (nm)**	**PDI**	**Zeta potential (mV)**	**Association efficiency (%)**	**Loading efficiency (% w/w)**
Loaded NC	261 ± 32	0.1 ± 0.03	+64 ± 3	92 ± 2	6.5 ± 0.14
Unloaded NC	196 ± 7	0.2 ± 0.03	+66 ± 2	—	
Loaded NE	164 ± 8	0.2 ± 0.02	–82 ± 8	50 ± 23	3.8 ± 1.75
Unloaded NE	167 ± 9	0.2 ± 0.05	–79 ± 3	—	
