# Supplementary material for: Chitosan encapsulation modulates the effect of capsaicin on the tight junctions of MDCK cells
Source: Sci Rep. 2015 May 13;5:10048. doi: 10.1038/srep10048 (PMC4429556; doi:10.1038/srep10048)
Supplement: Supplementary Information [file srep10048-s1.pdf]

Supplementary information:

Chitosan encapsulation modulates the effect of capsaicin on the tight junctions of MDCK cells

Kaiser M.[1], Pereira S.[1], Pohl L.[2], Ketelhut S.[2], Kemper B.[2], Gorzelanny C.[3], Galla H.-J.[4], Moerschbacher B.M.[1], Goycoolea F.M.[1*]

1: Institute of Plant Biology and Biotechnology (IBBP), Westfälische Wilhelms-Universität Münster, Schlossgarten 3, Münster 48149, Germany

2: Biomedical Technology Center of the Medical Faculty, Westfälische Wilhelms-Universität Münster Mendelstraße 17, Münster 48149, Germany

3: Experimental Dermatology, Department of Dermatology, Medical Faculty Mannheim, Heidelberg University, Theodor-Kutzer-Ufer 1-3, Mannheim 68167, Germany

4: Institute for Biochemistry, Westfälische Wilhelms-Universität Münster, Wilhelm Klemm Straße 2, Münster 48149, Germany

* goycoole@uni-muenster.de, Fax: +49 251 83-28371

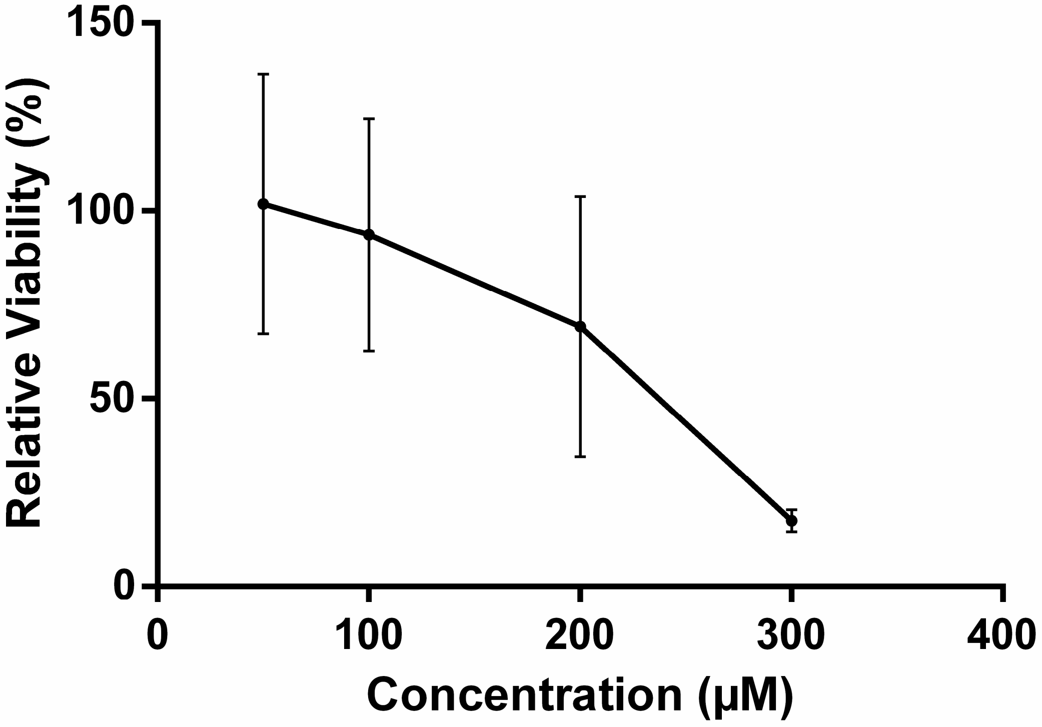

Cell Viability of MDCK-C7 cells after treatment with free capsaicin using DHM conditions (No CO_2 enriched atmosphere, HEPES buffer in medium). MTT assay for cell viability: Cells were incubated with capsaicin for 24 hours.
